# Mental health and macroeconomics: economic and social determinants that explain suicide in Ecuador

**DOI:** 10.3389/fpubh.2026.1701682

**Published:** 2026-02-23

**Authors:** Anderson Argothy, David Ortiz, Carolina García Ramos, Alba Vargas Espín

**Affiliations:** 1 Department of Economy, Faculty of Accounting, Technical University of Ambato, Ambato, Ecuador; 2 Department of Psychology, Faculty of Heath Sciences, Technical University of Ambato, Ambato, Ecuador

**Keywords:** behavioral economics, economic crisis, mental health, psychology, suicidal ideation, underemployment

## Abstract

**Background:**

Suicidal ideation and its most extreme outcome are important issues that should be studied from different theoretical approaches. Traditionally, it has been approached from the perspective of psychology; however, this study proposes an approach from the perspective of economics. The main objective of this study is to explain the effect and causality of economic and social determinants of suicide in Ecuador.

**Methods:**

The data for this study were collected from national and international sources and integrated to build a solid database that allows for this and other time series studies. Owing to the characteristics of the data, descriptive analysis and an econometric Vector Autoregressive (VAR) model were used. Stability tests were performed, and the Granger causality test was used to identify the causal directions of the variables.

**Results:**

The main results obtained using the model show that economic growth and underemployment significantly explain suicides.

**Conclusion:**

The study’s findings highlight the importance of developing mental health protection policies that consider social aspects and job security.

## 1 Introduction

Suicide represents a critical public health concern that has traditionally been examined through psychological and sociological lenses. However, recent advances highlight the importance of incorporating economic perspectives to better understand the underlying determinants of suicidal behavior (1, 2). From an economic analysis perspective, suicide is a relevant topic (1, 3–6). The interest is motivated by the fact that suicide rates are an indicator of satisfaction and quality of life, and related economic factors can be considered reasons for unhappiness (7). Traditionally, suicide has been studied from a theoretical sociological perspective, considering it a social phenomenon rather than a purely individual one, with rates varying according to the level of social integration, that is, individuals’ ties to the community, and moral regulation, understood as the norms that guide behavior (8).

In Ecuador, suicide remains a pressing issue, with limited research addressing its economic and social determinants. Existing studies have predominantly focused on psychological and sociodemographic factors, leaving a gap in understanding the macroeconomic and social influences on suicide within the country (9–11). In this sense, this research seeks to examine how economic and social determinants influence the suicide rate in Ecuador.

In behavioral economics, Gary Becker’s article is fundamental in addressing the choices individuals make. He considers the mechanisms of rational choice and their positions regarding death, life, marriage, and divorce (12). Furthermore, it challenges the traditional notion that agents always act rationally to maximize utility or profit without budgetary constraints. It argues that even when individuals exhibit irrational behavior—such as impulsivity or inertia that would explain suicide—their decisions are limited by sets of opportunities defined by resources, prices, and demand (5). In other words, although the individual act of suicide may seem irrational, aggregate patterns can be systematically affected by changes in individuals’ sets of opportunities.

The approach to suicide from an economic theory perspective differs from traditional mental health approaches. Economists argue that suicide should be studied as a rational decision influenced by economic and social factors, expectations of future wellbeing, and opportunity costs (3). Individuals compare the discounted present value of their expected lifetime utility to a critical threshold. If the expected utility falls below this threshold, suicide becomes a rational option (13). To develop this model, the following factors were considered: expected utility, consumption, living costs, age, and probability of survival. Based on this model, Marcotte (2003) extended it by considering suicide attempts as a probabilistic gamble, given that they are not always fatal, and that suicide attempts can alter future utility, as individuals may receive a transfer of resources or changes in health costs (3).

Rosenthal (14) introduces a game theory approach to rationalize suicide attempts that have non-trivial chances of success and failure, which he calls “playing with death.” From this perspective, suicide can be seen as a strategic interaction between an individual’s expectations and the possible responses of the environment (family, society, and health institutions). The attempt can have a double effect: on the one hand, the possibility of achieving the ultimate goal (death), and on the other, the possibility of failure, generating additional consequences such as hospitalization, family intervention, or changes in the social perception of the person. The attempt reveals a “high risk” type, which induces others to provide support; however, only credible attempts generate effective responses from others (14).

According to Prospect Theory (15), suicidal ideation is a choice made in the domain of extreme loss. Individuals who experience suicidal ideation often find themselves in a state of overwhelming psychological distress, perceiving their current situation as a series of unbearable losses or a generalized loss of wellbeing. Loss aversion states that losses are significantly more aversive than equivalent gains are attractive. In the context of suicidal ideation, it implies that the pain of perceived losses, such as hopelessness, isolation, and failure, is so intense that it drives a search for risk to escape them (15). Psychological theory considers perceived burden, that is, the belief that one’s own existence is a burden on others, as well as frustrated belonging, social disconnection, and loneliness, to be proximal factors for the desire to commit suicide (16).

These constructs can be reinterpreted as profound losses: perceived burden represents a fundamental loss of self-worth or social value, whereas frustrated belonging denotes the loss of vital social connections. These are not simply negative states but deeply aversive losses relative to an implicit benchmark of being valued and connected. The desire for suicide then becomes a risk-seeking behavior to escape these overwhelming perceived losses, even if the risk is one’s own life (15).

Efforts to explain the causes of suicide vary across different fields of science, with some attributing it to the use of alcohol, opioids, and other substances (17, 18). Suicide risk and personality disorders, in which certain diagnosed mental illnesses are identified as increasing the likelihood of suicide (19), were also considered. Localized studies in rural areas have shown that loneliness, climate change, and changes in family dynamics affect suicide rates (20).

There is a consensus in the literature that uncertainty drives economic cycles, making it a useful variable for analyzing the effects of socioeconomic factors on suicide (6). There is evidence that unemployment increases the risk of suicide, both due to loss of income and the damage it inflicts on self-esteem and self-perceived value. This impact can be particularly harmful to men, for whom employment is an important source of self-esteem (6, 21).

The relationship between economic growth and suicide is more complex and often contradictory. Although some studies have found a significant inverse relationship between income and suicide rates, others have reported a positive correlation (22, 23). Evidence suggests that rapid economic growth can be accompanied by social instability, which increases the risk of suicide. In contexts of high inequality and limited government support, GDP growth may be associated with additional pressure on working conditions (6, 21, 23, 24).

Regarding social factors, the literature shows that the risk of suicide increases with age, especially in men, and the loss of accumulated advantages in the labor market (high salaries, prestige) may be one reason for this. While unemployment affects both sexes, some studies suggest that men are more sensitive to macrosocial factors than women (21, 24). Female participation in the workforce has a notable effect on male suicide rates (21). Conversely, people with lower levels of education have a greater risk of suicide (24). However, during periods of economic crisis, educational differences in suicide mortality may decrease, reflecting the greater vulnerability of adults with medium-high educational attainment to economic changes, possibly due to loss of savings or the stigma of job loss in highly skilled careers (24). Income inequality can also increase the risk of suicide by generating economic discontent (24, 25).

Regarding suicide prevention, health spending is consistently associated with a reduction in male and female suicide rates, suggesting that a greater allocation of resources to health can translate into more services available to people at risk of suicide (21). However, suicide prevention requires a multilateral approach and is a legitimate function of the state and its institutions (4). It seeks to regulate, guide, and manage human behavior through practical knowledge, which allows for the participation of behavioral economics by considering the rationality of the individual. Suicide prevention policies incorporate political, economic, and institutional elements to curb the social and economic impacts of suicide and promote coordinated actions that increase the efficiency and quality of prevention services (4).

The logic of morality and political economy, being subjective, philosophical, and diverse within society, poses a problem for suicide prevention and care. A broad debate on morality may, on the one hand, consider certain behaviors of individuals to be “good.” At the same time, these behaviors may be classified as “bad.”

In political economic contexts, where the focus of power is on liberalization, privatization, decentralization, outsourcing, and other elements of the neoliberal doctrine, coordinating suicide prevention efforts can be complicated (4). If society, due to its political culture, is reluctant to understand arguments such as inequality in suicide mortality, it is very difficult to integrate effective prevention policies into the political agenda. An important way to highlight the relevance of suicide prevention is to focus on the costs associated with it and how it affects productivity and economic growth (4, 26, 27). This fundamentally considers the disparity of ethical frameworks, and the neoliberal vision focused on the benefits and costs associated with investing in prevention, disregarding equity, impartiality, and social justice. Under traditional economic logic, spending resources is more important than improving the quality of prevention policies or the management and provision of services.

In the Latin American context, there are few studies that analyze suicide and its social and economic determinants. Some have focused on education in Argentina (18); informal employment and symptoms of depression in Latin American cities (28); and sociocultural factors and suicidal behavior during COVID-19 (29, 30). In Ecuador, this is a latent problem, with an average of 1,047 suicides per year between 2007 and 2023, with an average of 797 women and 251 men. The burden of suicide in Ecuador (2001–2015) reported data on a suicide rate of 7.1 per 100,000 inhabitants, predominantly among men (11). Some studies examining this situation in the country focused on sociodemographic factors of older adults (9, 31), family issues, and suicide attempts in Cuenca (10).

This study aimed to evaluate the social and economic factors influencing the decision to commit suicide in Ecuador. This study contributes to the existing literature by including variables that have not been considered previously, as suicide in Ecuador has traditionally been approached from a psychological perspective. It also helps to close the existing gap in the social and economic characteristics of suicide in the country.

The remainder of this article is organized as follows. Section 2 describes the data and methodology used in this study. Section 3 presents the results of the study. Section 4 discusses the findings, and Section 5 draws conclusions and suggests future research directions.

## 2 Materials and methods

This study adopts a quantitative and explanatory research design to analyze the dynamic relationships between macroeconomic and social indicators and suicide mortality rates in Ecuador. The explanatory nature of this study goes beyond mere description or correlation, seeking to understand how and why certain economic and social factors influence suicides. The objective was to identify potential links and directions of influence, which align with the choice of a vector autoregressive (VAR) model. VAR models are particularly effective for structural inference and policy analysis in multivariate time series, as they allow for the examination of interdependencies between multiple variables as they change over time (32).

The geographical scope of this study is Ecuador. The period covered is from 2007 to 2023, and annual data is used. This time selection is crucial for time series analysis because it provides a sufficiently long horizon for robust econometric modeling.

The data for this study were obtained mainly from official national and international institutions to ensure reliability and comparability. The main sources include the World Health Organization (WHO), World Bank, and Ecuador’s National Institute of Statistics and Census (INEC). The variables chosen for this study were selected by the authors based on a review of the literature and the availability of data within the sources considered:

### 2.1 Suicide mortality

It is defined as the number of deaths reported as suicides annually. It is important to note that this is a crude value, meaning that it is not adjusted for age (33). Suicide mortality was chosen as a proxy because, although suicide mortality is a result and not the ideation itself, it represents the most serious and tragic manifestation of suicidal thoughts in each population. This variable has been used in previous studies either directly as an absolute value or as a mortality rate (6, 18, 21).

### 2.2 Economic growth

It refers to the change in the production of economic goods and services in one period compared to that in a previous period. It is measured by the annual percentage change in the Gross Domestic Product (GDP) at market prices. It is used as an indicator of economic activity and quality of life in countries (6, 21). It is a widely used variable for explaining social and economic phenomena of varying magnitudes.

### 2.3 Unemployment and underemployment

Ecuador’s employment surveys recognize several categories of occupations, of which two were considered for this study. Unemployment: According to the definition of the National Institute of Statistics and Census, persons aged 15 and over who were not employed during the reference period and who met certain characteristics are considered unemployed: (i) they did not have a job, were not employed in the previous week, and are available to work; (ii) they looked for work or took concrete steps to find employment or start a business in the previous 4 weeks (34). Underemployment: people with jobs who, during the reference week, earned less than the minimum wage and/or worked less than the legal working hours and were willing and available to work additional hours. It is the sum of underemployment due to insufficient working hours and income (34). The minimum wage is set annually, and for 2025, it is $470 USD per month, from which social security contributions are deducted. On the other hand, the working day is set at 8 h per day and up to 40 h per week. One of the characteristics of underemployment is that it generally involves precarious work, strenuous jobs, long hours, and low pay. Some studies that analyze the relationship between suicidal ideation and employment are provided in Refs. (28, 35, 36).

Unemployment and underemployment are distinct but related indicators of labor market health. While unemployment captures the total lack of employment, a high rate of underemployment, even with an apparently low official unemployment rate, suggests widespread economic precarity. This can be a significant stressor for individuals and households, as it implies insufficient income or job instability, which, in turn, can lead to financial stress.

The social variables selected in this study act as important indicators that can reflect underlying social stressors or directly contribute to individual distress. Based on a review of the literature, the following variables were selected:

### 2.4 Divorces

This variable represents the annual number of legally dissolved marriages in Ecuador (37). Gary Becker (12) stated that divorces are analyzed as an important indicator of social changes in family structure and associated psychological stress, such as emotional pain, financial instability, and social disruption, which are known risk factors for mental health problems. Some studies have analyzed this variable and its association with suicide (7, 17, 38).

### 2.5 Alcohol consumption

Total per capita alcohol consumption is defined as the total amount (sum of recorded and unrecorded alcohol) of pure alcohol consumed per person aged ≥ 15 years during a calendar year, adjusted for tourist consumption (39). Alcohol consumption can be seen as a maladaptive coping mechanism for underlying stress or as a direct contributor to mental health problems, disinhibition, and impulsivity, all of which can increase suicidal behaviors. Several studies have analyzed this social stressor in depth (16–19, 22).

By including these social variables, this study explored how changes in family stability and public health behaviors interact with economic conditions to influence the prevalence of suicide. This allows for a comprehensive view of social determinants.

Table 1 presents the descriptive statistics of the variables considered in this study.

**Table 1:** Descriptive statistics.

**Variable**	**Obs**	**Mean**	**Std. Dev.**	**Min**	**Max**
*suic*	17	1.047,12	1.689,47	694,00	1.233,00
*growth*	17	3,24	4,50	–9,25	9,82
*enemp*	17	3.896,77	0,74	3.083,00	6.116,00
*underemp*	17	1.675,29	4,30	9,00	23,20
*div*	17	21.933,06	4.311,21	14.568,00	28.771,00
*alc*	17	3,56	0,56	2,70	4,19

### 2.6 Methods

The VAR model allows complex interdependencies to be captured without imposing strong theoretical restrictions on the relationships between the variables. In a VAR system, each variable is modeled as a linear function of its own lagged values and the lagged values of all other variables in the system. This flexibility makes VAR models particularly useful for describing the dynamic behavior of economic and social time series (32, 40). For Ecuador, this model allows us to examine how the economic cycle (growth, unemployment, and underemployment) interacts with social variables (divorce and alcohol consumption) and their impact on suicide mortality.

The structural form of the model is as follows in Equation 1:


(1) $$Y_{t}=A_{0}+\sum_{i=1}^{p}A_{1}Y_{t-1}+U_{t}\;\;\;\;$$

where:

$Y_{t}={\left[\mathrm{sui}c_{t},\text{growt}h_{t},\text{unem}p_{t},\text{underem}p_{t},\mathrm{di}v_{t},\mathrm{al}c_{t}\right]}^{\prime }$ is a 6×1 vector.

$A_{0}$ is a vector of intercepts.

$A_{i}$ 6 × 6 matrices of coefficients.

$U_{t}$ vector de errores con $E[U_{t}]=0$ and covariance matrix $\Sigma_{u}$ .

The variables in the model are as follows:

suic : Total annual suicide.

growth : Economic growth.

unemp : Unemployment rate.

underemp : Underemployment rate.

div : The annual number of divorces.

Given the analysis period, some variables may show trends or non-stationarity. Dickey–Fuller and Phillips–Perron unit root tests were applied (32) to each series (Table 2). The integrated variables of order one were included in the model by applying the first differences. Several information criteria were used to select the optimal lag length (Table 3), such as the Akaike Information Criterion (AIC), Bayesian Information Criterion (BIC), and Hannan–Quinn Information Criterion (HQIC) (32, 41). This avoids over-parameterization. Since the data series is small, after performing the optimal lag test, the variables *unemp* and *growth* were removed to improve the fit of the data.

**Table 2:** Phillips–Perron test for unit root.

Phillips–Perron test for unit root Interpolated Dickey-Fuller								
	**Test statistic**	**1% Critical value**	**5% Critical value**	**10% Critical value**	**MacKinnon approximate *p*-value for Z(t)**	**Number of observation**	**Newey-WestLags**	**Stationary**
d_suic								
*Z*(rho)	−11.988	−17.200	−12.500	−10.200	0.0136	15	2	Yes
*Z*(t)	−3.330	−3.750	−3.000	−2.630				
d_underemp								
*Z*(rho)	−11.886	−17.200	−12.500	−10.200	0.0286	15	2	Yes
*Z*(t)	−3.073	−3.750	−3.000	−2.630				
d_div								
*Z*(rho)	−11.988	−17.200	−12.500	−10.200	0.0000	15	2	Yes
*Z*(t)	−3.330	−3.750	−3.000	−2.630				
d_alc								
*Z*(rho)	−7.933	−17.200	−12.500	−10.200	0.1971	15	2	No
*Z*(t)	−2.226	−3.750	−3.000	−2.630				

**Table 3:** Lag selection order.

Selection-order criteria								
Sample: 2010–2023				Number of obs = 14				
lag	LL	LR	df	*p*	FPE	AIC	HQIC	SBIC
0	−257.274				2.8e+12*	37.1819*	37.1693*	37.3189*
1	−251.966	10.615	9	0.303	5.0e+12	377.094	376.587	382.572
2	−244.496	14.94	9	0.093	8.0e+12	37.928	378.393	388.866
Endogenous: d_suic d_underemp d_div								
Exogenous: _cons								

Several models were estimated, including all the variables considered above. However, after autocorrelation tests, it was decided that the best model is the one proposed in the equation:


(2) $$\begin{array}{l} \mathrm{sui}c_{t}=\beta_{1,0}+\beta_{1,1}\mathrm{sui}c_{t-3}++\beta_{1,2}\text{underem}p_{t-3}\\ +\beta_{1,3}\mathrm{di}v_{t-3}+u_{1t}\;\; \end{array}$$

The study focuses on and resolves using Equation 2, the VAR method allows for the evaluation of the multiple directions of the variables and their effect as dependent and independent variables.

Once the model was estimated, the autocorrelation test, the LM heteroskedasticity test (Table 4), the impulse response test (Table 5), and the Granger causality analysis (Table 6) were performed (32, 40) to identify significant predictors and the direction of causality between the variables.

**Table 4:** Autocorrelation test and heteroskedasticity.

(a) Lagrange-multiplier test			
**lag**	**chi2**	**df**	**Prob > chi2**
1	78.878	9	0.54549
2	60.940	9	0.73048
H0: no autocorrelation lag order			

(b) LM test for autoregressive conditional heteroskedasticity (ARCH)			
lags(p)	chi2	df	Prob > chi2
1	1.123	1	0.2893
2	1.485	2	0.4758
H0: no ARCH effects vs. H1: ARCH(p) disturbance			

**Table 5:** Impulse response of suicide.

Results from IRF			
**Step**	**(1) irf**	**(2) irf**	**(3) irf**
0	1	0	0
1	−0.053732	259.681	0.009094
2	−0.402702	302.345	0.007624
3	0.247688	−759.857	−0.013176
4	0.138438	−162.541	−0.001351
5	−0.248644	268.102	0.006955
6	−0.046211	479.035	−0.001075
7	0.17539	−444.396	−0.003851
8	−0.010697	−0.918516	0.002108
9	−0.103423	428.846	0.002002
10	0.038477	−0.135491	−0.001994

(1) irfname = ImpRep, impulse = d_suic, and response = d_suic. (2) irfname = ImpRep, impulse = d_underemp, and response = d_suic. (3) irfname = ImpRep, impulse = d_div, and response = d_suic.

**Table 6:** Granger causality Wald tests.

**Equation**	**Excluded**	**chi2**	**df**	**Prob > chi2**	**Causality**
d_suic	d_underemp	13.972	2	0.001	Yes
d_suic	d_div	52.628	2	0.072	Yes
d_suic	ALL	14.782	4	0.005	The set of excluded variables is significant
d_underemp	d_suic	46.882	2	0.096	Yes
d_underemp	d_div	0.75042	2	0.687	No
d_underemp	ALL	47.739	4	0.311	The set of excluded variables is not significant
d_div	d_suic	0.64741	2	0.723	No
d_div	d_underemp	19.731	2	0.373	No
d_div	ALL	20.486	4	0.727	The set of excluded variables is not significant

## 3 Results

This section presents the results of applying the methodology described in the previous section. Table 2 shows the stationarity test of the variables to avoid spurious results in the model.

Table 3 shows the lag selection order criteria. Considering the size of the time series and the optimal lag test, the model was estimated using two lags to increase the robustness of the model, and it can be tried with 1 or 2 lags (42).

Table 7 presents the vector autoregressive (VAR) estimates of the variables considered.

**Table 7:** Estimation with VAR regression model.

Sample: 2011–2023					Number of obs = 14
Log likelihood = −244.4959					AIC = 37.92799
FPE = 8.00e+12					HQIC = 37.83926
Det(Sigma_ml) = 2.96e+11					SBIC = 38.88658
**Equation**	**Parms**	**RMSE**	***R*-sq**	**chi2**	**P > chi2**
d_suic	7	113.429	0.6007	2.106.441	0.0018
d_underemp	7	302.171	0.3302	6.902.678	0.3299
d_div	7	5184.27	0.2870	5.635.152	0.4653

		**Coef.**	**Std. Err.**	***z***	**P > |z|**	**[95% Conf. Interval]**	
d_suic	*d_suic*						
	L1.	−0.053732	0.1797192	−0.30	0.765	−0.4059752	0.2985111
	L2.	−0.6315297	0.182479	−3.46	0.001	−0.989182	−0.2738773
	d_underemp						
	L1.	2.596.806	10.692	2.43	0.015	5.012.124	46.924
	L2.	2.315.643	9.274.166	2.50	0.013	4.979.396	4.133.346
	d_div						
	L1.	0.0090944	0.006473	1.40	0.160	−0.0035925	0.0217814
	L2.	0.0139565	0.0062101	2.25	0.025	0.0017849	0.026128
	_cons	−9.939.151	2.329.095	−0.43	0.670	−5.558.858	3.571.028
d_underemp	d_suic						
	L1.	0.0102695	0.0047877	2.15	0.032	0.0008859	0.0196532
	L2.	−0.0015301	0.0048612	−0.31	0.753	−0.0110578	0.0079976
	d_underemp						
	L1.	0.181833	0.2848312	0.64	0.523	−0.3764259	0.7400919
	L2.	−0.3235247	0.2470606	−1.31	0.190	−0.8077545	0.1607051
	d_div						
	L1.	−0.0000879	0.0001724	−0.51	0.610	−0.0004259	0.0002501
	L2.	−0.0001412	0.0001654	−0.85	0.393	−0.0004654	0.0001831
	_cons	0.2289787	0.6204629	0.37	0.712	−0.9871064	1.445.064
d_div	d_suic						
	L1.	−4.479.599	8.214.059	−0.55	0.586	−2.057.886	1.161.966
	L2.	−4.892.431	8.340.197	−0.59	0.557	−2.123.892	1.145.405
	d_underemp						
	L1.	412.504	4.886.775	0.84	0.399	−5.452.862	1.370.294
	L2.	4.246.564	4.238.754	1.00	0.316	−406.124	1.255.437
	d_div						
	L1.	−0.3916296	0.2958504	−1.32	0.186	−0.9714857	0.1882264
	L2.	0.0600752	0.2838311	0.21	0.832	−0.4962236	0.616374
	_cons	4.052.415	1.064.512	0.38	0.703	−1.681.164	2.491.647

Table 4 shows the autocorrelation test, and the LM test for heterskedasticity, to avoid problems in the estimation model.

The impulse response analysis of the suicide variable over time is shown in Table 5.

The direction of causality of the model variables was tested using Granger’s causality test (Table 3).

The unit root test of the variables (Appendix 1) shows that two of the six selected variables are stationary (*growth, unemp*). In this regard, first differences were applied to the variables (*suic, undermp, div, alc*) (Table 2). Stationarity was achieved with the new variables except in *d_alc*, and the model was estimated.

Considering the size of the time series and the optimal lag test (Table 3), the model was estimated using two lags to increase the robustness of the model, and it can be tried with 1 or 2 lags (42). Table 7 presents the results of the proposed VAR model estimation. The variable per capita alcohol consumption (alc) was not included in the model as it presented problems and affected the stability of the model; *growth, unemp* was not included to avoid autocorrelation due to the short time series. Three models were estimated, one for each variable. A characteristic of this method is that the variables are dependent and independent, which in some cases allows for the identification of bidirectionality in causality.

Each equation has seven parameters. The *R*-squared ranges from 0.287 to 0.601, and the joint significance tests are *p* < 0.05, indicating that the lags of the variables together explain a substantial fraction of the contemporary variation.

The coefficients of the equation explaining suicide highlight the autoregressive effect of suicide. L2 *p* < 0.05 with a negative sign suggests a strong reversion to the mean, increases, or decreases that tend to correct themselves in the two subsequent periods. This shows the dominant dynamics of the phenomenon. The adjustment is channeled via underemployment or other dimensions of precariousness that better capture vulnerability. One of the characteristics of the Ecuadorian economy is high underemployment that hides real unemployment (43, 44). The underemployment coefficients show L1 and L2 (*p* < 0.05); the positive sign at the first lag indicates that the immediate precariousness of the underemployment situation causes an increase in suicides. This result expands on the study of Skinner et al. (45), who found a correlation between suicide and underemployment in Australia. Over a longer time horizon, it becomes a buffering variable that reduces suicides in Ecuador. The social factor of divorce was significant in L2 (*p* < 0.05) with a positive sign, which means that the increase in divorces, probably due to the emotional impact and the economic and social situations following the end of the marital relationship, leads to the decision to commit suicide. This result confirms the findings of (38), who identified that divorce increases the risk of suicidal ideation in Sweden.

The other blocks in Table 7 show how social and economic factors affect underemployment and divorce rates in Ecuador. In this study, Granger causality was used to analyze the existence of bidirectionality (Table 6).

Appendix 2 shows the results of these stability tests. Values below one and all points within the circle indicate that the VAR is stable, meaning that shocks to the variables are transitory; that is, a significant variation in the variable has a dynamic effect that dissipates over time. To determine the validity of the estimated model, robustness tests were performed (Tables 4a,b). The results obtained (*p* > 0.05) in the two tests confirm that there are no problems of autocorrelation or heteroscedasticity, so the model has been correctly estimated with the proposed regressors. This strengthens the results found in this study.

Table 5 of impulse-response functions describes the variation in the suicide rate (d_suic) in response to three types of shocks: one specific to the individual, underemployment, and divorce dynamics. This table shows the dynamic approach of the VAR model over 10 periods. The response of suicides to an impulse in underemployment is the most relevant relationship from an economic and mental health perspective. In the first period after the shock (*t* = 1), the response is positive and large (25.97), intensifying in the second period (30.23), before reversing to negative values at *t* = 3 (−7.60) and *t* = 4 (−16.25). From *t* = 5 onwards, the effect loses strength and fluctuates around zero.

The response of suicides to a surge in divorces is very small (in the order of thousandths) and does not maintain a clear direction: the first effect is slightly positive (0.009 at *t* = 1), followed by values that alternate in sign between *t* = 2 and *t* = 10.

Table 6 presents a summary of Granger causality, which allows us to understand whether there is self-reinforcement between variables when they go from being explained to explanatory. In the first block, the variables underemployment (*p* = 0.01) and divorce (*p* = 0.07) significantly caused or predicted the number of suicides in Ecuador with a *p* < 0.10. Although there is bidirectionality between suicide and underemployment, there is a significant causality (*p* = 0.096) in explaining underemployment, and there is almost perfect bidirectionality between these two variables (*p* < 0.10). Suicide does not cause divorce, as its value is not significant (*p* = 0.723), indicating unidirectionality between the variables. Taken together, each equation can be explained using the variables included in the model.

**Figure 1:**
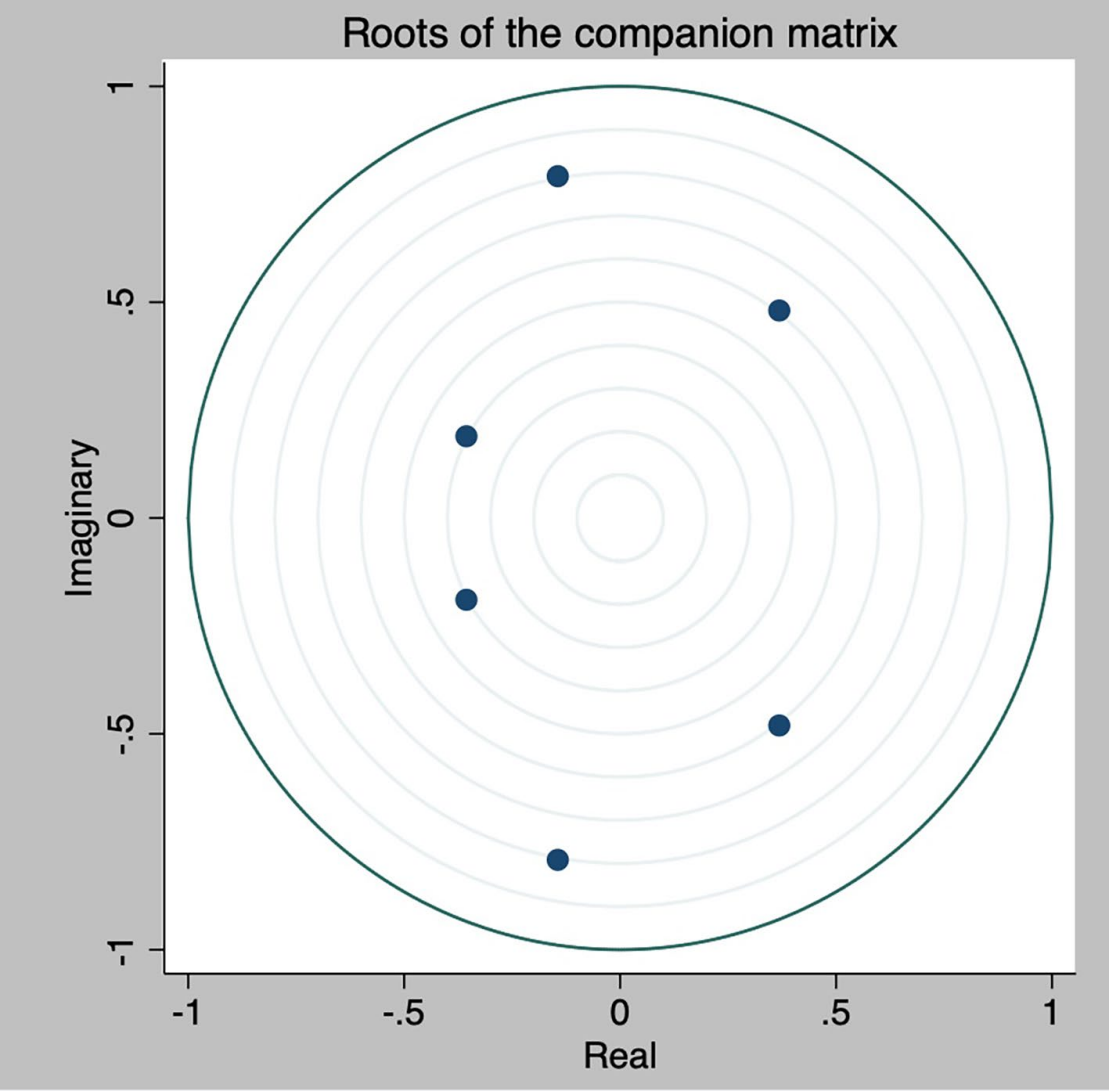
Stability graph.

Individual analyses of each equation are possible; however, this study primarily aimed to explain the social and economic factors of suicide in Ecuador. Therefore, it does not delve into other relationships (Figure 1).

## 4 Discussion

The analysis of the economic and social determinants of suicide in Ecuador using the Vector Autoregressive (VAR) model reveals several important and nuanced insights into the complex dynamics underlying suicidal behavior within the country. The stationarity tests conducted on the data confirmed the necessity to transform certain variables through first differences to ensure the robustness and validity of the econometric modeling.

The application of time series and VAR models allows us to identify the economic (underemployment) and social (divorce) factors that explain suicide in Ecuador. Changes in these variables, considering the lags analyzed, had significant effects on suicide. However, despite the model tests carried out, the limited number of observations (*n* = 17) imposes restrictions on inferences, which should be interpreted with caution by the reader. Parameter estimators may be biased in infinite samples. In this case, the lags used should be interpreted as preliminary evidence of contemporary and short-term dynamic associations in the Ecuadorian context, rather than as stable long-term structural parameters. According to the literature, economic factors affect suicidal ideation (28, 35, 45, 46). Social factors, specifically divorce, have not been extensively studied as factors influencing suicide in Latin America (38). This study shows that there is a significant causality in the Ecuadorian case.

It is important to mention that aggregate suicide data do not capture the demographic heterogeneities documented in the international literature. Robust evidence indicates that the relationship between economic factors and suicide varies significantly by age group and gender (9, 11). For example, in contexts similar to Ecuador, unemployment affects working-age men more severely, while underemployment and precariousness have a greater impact on young women (34). Although the proposed model could not include this disaggregation due to limitations in access to official historical series, the estimated effects should be interpreted as population averages that may mask specific dynamics.

Macroeconomic disturbances, such as economic recessions, fluctuations in GDP growth, and labor market instability, combined with fragile working conditions characterized by precarious employment, low wages, and insufficient working hours, serve as significant triggers for suicidal behavior in Ecuador (6, 26). These factors create an environment of economic uncertainty and financial stress that disproportionately affects vulnerable populations, exacerbating feelings of instability and psychological distress (1, 47). The persistent presence of underemployment in Ecuador, which masks true labor market hardships, further intensifies this vulnerability by limiting individuals’ ability to secure stable and adequate income (45, 48). Consequently, it becomes imperative to implement comprehensive social protection policies and employment measures aimed at cushioning these populations from the adverse effects of economic shocks. Such interventions should focus on improving job security, promoting formal employment, ensuring fair wages, and providing access to mental health and social support services. By addressing both the economic and social dimensions of vulnerability, these measures can mitigate the risk factors that lead to suicidal behavior and contribute to enhanced overall wellbeing and adaptation in the face of economic challenges. The unexpected increase in underemployment causes an immediate and significant rise in suicide rates, an effect that lasts for a short period and then dissipates (45). Divorces do not generate a clear or sustained response in suicides. The relationship between family breakdown and suicidal behavior could exist in population subgroups or manifest itself over longer periods, but no significant transmission is observed in the short term modeled (38). The bidirectional or feedback relationship between the economic cycle and suicide mortality not only confirms that economic recessions, captured by economic variables, precede and predict increases in suicide rates (7, 23), but there is also a reverse effect: changes in the suicide rate Granger-cause changes in underemployment. This finding suggests that the deterioration of the population’s mental health, the most extreme indicator of which is suicide, is a drag on economic development due to the loss of human capital, decreased labor productivity, and increased health care costs. This bidirectionality highlights that the relationship between the economy and mental health is complex and synergistic.

This situation reveals a vicious circle of negative feedback with socioeconomic implications. On the one hand, underemployment acts as a chronic stressor that deteriorates mental health and increases the risk of suicide. Conversely, the prevalence of mental health problems associated with suicide reduces productivity, increases absenteeism, and erodes human capital, affecting local competitiveness and perpetuating job insecurity (27, 28, 35). In addition, suicides generate significant social costs, loss of productivity, and damage to community cohesion, factors that together weaken the foundations for inclusive economic development (24, 46). This finding underscores the urgency of designing integrated employment and mental health policies that break this cycle, particularly in informal economies and vulnerable communities.

Divorce, a stressful event that impacts individuals’ financial stability and emotional wellbeing, is a powerful predictor of extreme personal crises (suicide), highlighting the critical importance of incorporating social cohesion variables into econometric models that study these phenomena. The social determinant of divorce stands out as a significant factor positively associated with suicide rates at the second lag. The emotional turmoil, financial instability, and social disruption following marital dissolution likely contribute to heightened psychological distress and increased vulnerability to suicidal behavior.

Mental health should be prioritized in Ecuador. The significant results of the variables studied show that citizens find it difficult to cope with social and economic stress. It is essential to develop and implement suicide prevention policies that consider the social impacts of underemployment and economic crises, as well as other situations such as divorce or alcohol and substance abuse. Furthermore, support should not be limited to the short term but should also be provided in the medium and long term.

## 5 Conclusion

This study shows how underemployment and divorce in Ecuador are stressors that contribute to the deterioration of mental health, which can lead people to commit suicide. A VAR model was used for this study and estimated with two lags.

The results of this study suggest the need to integrate policies promoting formal employment and family care services as suicide prevention strategies in the country. It is necessary to move beyond the health sector’s approach to suicide prevention and extend it to other areas, such as the economic and family spheres. State institutions must develop public policies to improve the quality of life in order to reduce the loss of lives to suicide due to economic causes or family breakdown. The feedback relationships between the variables show the need for public policy to take a medium- and long-term approach to reducing suicide rates.

This study highlights the existence of a vicious cycle between underemployment and suicide, where each factor feeds into the other, exacerbating both job insecurity and mental health crises. This bidirectional relationship suggests that public policies should not address these problems in isolation. Therefore, it is recommended to include integrated intersectoral strategies that combine quality job creation with community mental health interventions, especially in areas most affected by informal employment and psychosocial vulnerability.

Within public policy, the findings of this study support the implementation of integrated interventions that combine social protection and mental health. In the Ecuadorian context, the social transfer program known as the “Human Development Bonus” could evolve into a scheme conditional not only on school attendance or health but also on participation in technical training programs accompanied by psychosocial support, especially in households affected by divorce or long-term unemployment. The National Mental Health Strategy should formalize operational links with the Ministry of Labor to implement mental health-focused employment counseling in Employment Offices. These evidence-based measures could mitigate the vicious cycle identified, translating the findings of the model into concrete prevention actions.

The main limitation of this study was the lack of more extensive historical data that would allow for a better understanding of the effect of time on the factors that affect suicide in Ecuador, and the exclusion of clinical mental health variables. A significant methodological limitation was the exclusion of theoretically relevant variables, such as alcohol consumption, due to stationarity problems that persisted even after applying logarithmic and difference transformations. Alternative specifications were explored (VAR in first differences with I(1) variables), but their inclusion generated instability in the roots of the model and affected the robustness of the results. This omission may restrict the explanatory scope of the study, given the consensus in the literature on alcohol as a risk factor in suicidal behavior (17, 18).

For future research, it is recommended that panel studies be conducted in Latin America, Europe, and Asia. This will contribute to scientific knowledge in the region and the development of prevention policies based on empirical studies using real data.

## Data Availability

The original contributions presented in the study are included in the article/Supplementary material, and further inquiries can be directed to the corresponding author/s.

## Appendix 1

**Table:**

Phillips–Perron test for unit root								
Interpolated Dickey–Fuller								
								
	**Test statistic**	**1% Critical value**	**5% Critical value**	**10% Critical value**	**MacKinnon approximate *p*-value for Z(t)**	**Number of observation**	**Newey-West Lags**	**Stationary**
suic								
Z(rho)	−4.224	−17.200	−12.500	−10.200	0.5840	16	2	No
Z(t)	−1.396	−3.750	−3.000	−2.630				
growth								
Z(rho)	−15.865	−17.200	−12.500	−10.200	0.0023	16	2	Yes
Z(t)	−3.860	−3.750	−3.000	−2.630				
unemp								
Z(rho)	−11.774	−17.200	−12.500	−10.200	0.0405	16	2	Yes
Z(t)	−2.944	−3.750	−3.000	−2.630				
underemp								
Z(rho)	−7.696	−17.200	−12.500	−10.200	0.6445	16	2	No
Z(t)	−2.506	−3.750	−3.000	−2.630				
div								
Z(rho)	−7.696	−17.200	−12.500	−10.200	0.1140	16	2	No
Z(t)	−2.506	−3.750	−3.000	−2.630				
alc								
Z(rho)	−0.151	−17.200	−12.500	−10.200	0.9485	16	2	No
Z(t)	−0.110	−3.750	−3.000	−2.630				

## Appendix 2

**Table:**

Eigenvalue stability condition	
**Eigen value**	**Modulus**
−0.1443682 + 0.791861i	0.804914
−0.1443682 – 0.791861i	0.804914
0.3684929 + .4806127i	0.60562
0.3684929 – 0.4806127i	0.60562
−0.355889 + 0.189446i	0.403171
−0.355889 – 0.189446i	0.403171

All the eigenvalues lie inside the unit circle. VAR satisfies stability condition.
